# Effects of Urban Landscape Pattern on PM_2.5_ Pollution—A Beijing Case Study

**DOI:** 10.1371/journal.pone.0142449

**Published:** 2015-11-13

**Authors:** Jiansheng Wu, Wudan Xie, Weifeng Li, Jiacheng Li

**Affiliations:** 1 The Key Laboratory for Environmental and Urban Sciences, School of Urban Planning and Design, Peking University Shenzhen, Shenzhen, China; 2 College of Urban and Environmental Sciences, Laboratory for Earth Surface Processes, Ministry of Education, Peking University, Beijing, China; 3 Department of Urban Planning and Design, University of Hong Kong, Hong Kong, China; 4 Department of Urban Development and Land Policy, Urban Planning & Design Institute of Shenzhen, Shenzhen, China; Tsinghua University, CHINA

## Abstract

PM_2.5_ refers to particulate matter (PM) in air that is less than 2.5μm in aerodynamic diameter, which has negative effects on air quality and human health. PM_2.5_ is the main pollutant source in haze occurring in Beijing, and it also has caused many problems in other cities. Previous studies have focused mostly on the relationship between land use and air quality, but less research has specifically explored the effects of urban landscape patterns on PM_2.5_. This study considered the rapidly growing and heavily polluted Beijing, China. To better understand the impact of urban landscape pattern on PM_2.5_ pollution, five landscape metrics including PLAND, PD, ED, SHEI, and CONTAG were applied in the study. Further, other data, such as street networks, population density, and elevation considered as factors influencing PM_2.5_, were obtained through RS and GIS. By means of correlation analysis and stepwise multiple regression, the effects of landscape pattern on PM_2.5_ concentration was explored. The results showed that (1) at class-level, vegetation and water were significant landscape components in reducing PM_2.5_ concentration, while cropland played a special role in PM_2.5_ concentration; (2) landscape configuration (ED and PD) features at class-level had obvious effects on particulate matter; and (3) at the landscape-level, the evenness (SHEI) and fragmentation (CONTAG) of the whole landscape related closely with PM_2.5_ concentration. Results of this study could expand our understanding of the role of urban landscape pattern on PM_2.5_ and provide useful information for urban planning.

## Introduction

PM_2.5_, which refers to particulate matter (PM) in air that is less than 2.5μm in aerodynamic diameter [1], is a key pollutant affecting human health, visibility and radiation balance [2]. The small size, strong adsorption and complex constitution are major features, as it can carry heavy metals and sulfates, etc. into the respiratory tract and lungs [3]. PM_2.5_ mainly originates from the products of our daily activities, such as vehicle exhaust, marine aerosols, coal and fuel oil combustion, burning of agricultural wastes, paved road dust, and secondary sulfates, etc. [4]. Owing to population expansion and rapid urbanization, PM_2.5_ has become one of the most serious environmental problems in many cities in China [5, 6]. Big cities, such as Beijing and Shanghai, have suffered terrible hazes in recent years, causing many environmental and ecological problems, and also making troubles in human travel [1, 7], and is drawing more and more attention around the world. This problem is especially urgent in China, which is experiencing rapid urbanization and is planning to continue giving high priority to urbanization in the coming decades [8].

PM_2.5_ pollution is a main factor in decreasing air quality. It not only causes serious visibility problems, but also does great harm to human health. First, PM_2.5_ can impair visibility by light scattering through its suspended particles and gases, especially its components such as primary and secondary organics, inorganic salt, inorganic carbon, and elements [9]. Second, long-term exposure to PM_2.5_ can result in mortality and morbidity [10]. Previous studies have confirmed that high PM_2.5_ concentration is associated with increased rates of cardiovascular and respiratory diseases [11], and also leads to cancer, as well as premature death [12]. Last, PM_2.5_ can affect Earth’s radiation balance and influence energy balance and material flow [4]. Thus, how to decrease PM_2.5_ concentration to reduce its adverse impact has become a hot issue among researchers.

Recent studies have mainly focused on source appointment [6], measurement and simulation [13] and health risk assessment of PM_2.5_ [14], attempting to make clear the chemical components and sources and then try to find out some effective measures to reduce the negative effects. Additionally, many studies have explored the relationship between air pollution and land use [15–17]. Besides, the changes of landscape patterns, which includes the changes of composition and configuration of landscape can influence PM_2.5_. Furthermore, various methods have been explored and applied to research the factors of PM_2.5_ concentrations. Regression analysis is a common method among them[18], especially the Land Use Regression model (LUR) [19–21], which analyzes the potential factors based on GIS. Traffic, winter heating, farm burning, and land use, etc. are found to greatly influence PM_2.5_. Take the ESCAPE project [22] in Europe as an example, it used LUR to explore pollutant sources, including factors such as road networks, population density, land use, and elevation (DEM), etc. Besides, a number of studies also concentrate on the effects of specific land use types, such as vegetation, water etc., on PM_2.5_. The relationship between vegetation and air pollutant was a common focus in experiment and simulation studies. Sabo et al. [23] examined the PM accumulation on leaves of 22 trees and 25 shrubs in test field in Norway and Poland. The results showed that Pinus mugo and Pinus sylvestris, Taxus media and Taxus baccata, Stephanandra incisa and Betula pendula were efficient species in capturing PM. Broad-leaved species with rough leaf surfaces are more efficient in capturing PM than those with smooth leaf surfaces [24]. Modelling approaches to research the PM deposition to the urban tree canopy were conducted in Japan [25], London [26] and New York [27] et al. Green land was usual variable in land-use regression models [28, 29]. As for water, winds carry billions of tons of PM from the continents to the oceans. Marine and atmospheric scientists were investigating the transport and deposition of Pm to the ocean. The flux of PM depends on many factors including the distribution of sources, the physical and chemical properties of the PM, meteorological conditions, and the rates of removal by dry and wet deposition. Atmospheric chemical transport models, coupled with in situ observations, were improving our understanding of the temporal and spatial variability of PM deposition to oceans [30]. ESCAPE case study in Stockholm County also found that water in buffer of 500 meters had negative effect on PM_2.5_ concentration [22]. These studies all displayed the reducing influence of vegetation and water on particulate matter.

The relationship between land use and PM_2.5_ has been confirmed in previous studies, but there is not enough information offered in these studies to explore the effects of urban landscape patterns on PM_2.5_ pollution. The relationship between urban landscape patterns and air pollution is a complex patterns-process relationship. Recently, with the development of RS and GIS, research has started to focus on the influence of urban form and urban landscape on air pollution. Tang and Wang [31] demonstrated that urban form had effects on traffic-induced noise and air pollution, such as urban forms in historical area with narrower roads, complex road network led to lower noise pollution, but the greater street canyon effects results in higher CO concentration. Weber et al. [32] revealed that landscape metrics in different urban structures were good indicators of PM_10_ without measured data, which indicated the function of urban landscape on air pollution. An study case of evaluating how spatial heterogeneity of forest affect air pollution was conducted by Escobedo and Nowak [33], which also took urban landscape of green space into account. As for the relative studies in China, there were also some papers proving the importance of landscape patterns in green space on air quality [34]. The fragmentation of green space has been shown to affect the concentration of fine particulate matter in Yichang city [35]. By means of calculating and analyzing several landscape metrics, these studies investigate how to make a better landscape planning to reduce air pollution, such as how to plan vegetation to deposit PM_2.5_ more efficiently [36]. The reason that landscape pattern influence PM_2.5_ concentration may depend on many factors. The landscape could firstly change the factors, such as transportation volume and wind trace and further influence the air pollutant concentration. Moreover, different landscape pattern perhaps affected the interaction between forest, water and particulate matter in the air.

However, compare to noise pollution [31, 32], water pollution [37–39] and other ecological processes [40], there is little research focusing on the relationship between air pollution, especially fine particulate matter and urban landscape patterns [33, 34]. More attention has been paid to the influence of different land cover on air pollution, rather than different landscape pattern. In addition, it is hard to explain the mechanism of the urban landscape pattern on particulate matter though it may have some similar process with heat island. The other possible reasons for rare studies may include the appropriateness of method, limitation of data, etc. [41, 42]. As a result, firstly capturing the quantitative relationships between landscape patterns and PM_2.5_ is of theoretical importance and practical for optimizing urban landscape patterns and improving air quality in the environment, especially in China today. Furthermore, it can widen our understanding of the relationship between landscape patterns and ecological process and its effects on air pollution.

Urban landscapes are characterized by complex spatial heterogeneity, as different land-cover and land-use types have their own surface characteristics. Landscape metrics are algorithms that quantify specific elements [32] and spatial characteristics, including patches, classes, and entire landscapes, and are usually used in urban form and urban landscape research [41]. Further, landscape metrics are also applied to investigate the influence of composition and configuration of different land use types on biodiversity [43] and habitat [44]. Additionally, they can be calculated quickly and directly. Thus, it is appropriate to use landscape metrics to investigate the effect of urban landscape patterns on PM_2.5_ concentration.

The main objective of this paper is to examine the effects of urban landscape pattern on PM_2.5_ pollution. The study site is Beijing, China’s capital, which has limited green space [13] and has been experiencing serious hazes in recent years [21]. Therefore, the results of this study can contribute to improving urban landscape planning and management, and can be as effective measures for addressing air quality problems in Beijing. We used variables including PM_2.5_ concentration, street networks, elevation, population and landscape metrics to build regression models of all year and each season. Then we investigated the effects of urban landscape patterns on PM_2.5_ pollution in Beijing through statistical analysis. Specifically, the study addressed the following questions:

1. How do urban landscape composition affect PM_2.5_ concentration?
2. How do urban landscape configuration affect PM_2.5_ concentration?
3. How do whole landscape pattern affect PM_2.5_ concentration?

## Study area

Beijing is located in the northeast of the North China Plain (115°25′-117°30′E, 39°28′-41°25′N) with a population of 20.693 million and a total area of 16410.54 km^2^. Its climate belongs to a continental monsoon climate with apparent seasons. The average temperature is 12.3°C, and annual precipitation is about 572mm [45]. We used MAM (March, April and May), JJA (June, July, and August), SON (September, October, and November), and DJF (December, January, and February) to represent spring, summer, autumn, and winter respectively. As a city with a history of more than 3000 years, Beijing has served as the capital for more than 850 years [46]. Since the implementation of China’s reform and opening-up policy in 1978, Beijing has been undergoing rapid urbanization. Construction land area and traffic volume has increased dramatically, which has resulted in severe air pollution. Frequent haze events and high pollutant concentrations in this city have attracted the attention of the world. Additionally, along with urbanization, there is less impossible to increase area, especially of green space, which can mitigate the PM_2.5_ concentration [31]. So how to make use of the landscape pattern to improve air quality in limited areas is of great significance for sustainable development in Beijing.

## Data and methods

To explore the effects of landscape patterns on PM_2.5_ concentration, the following steps were taken: (1) PM_2.5_ concentration of 35 monitoring sites in Beijing was obtained from the website of the Beijing Environmental Monitoring Center; (2) relevant variables, including street network, population, and elevation etc. were analyzed using GIS; (3) a selection of landscape metrics were calculated in Fragstats, including class level and landscape level, to provide information about features of landscape composition and configuration; (4) statistical analysis, mainly including Pearson’s correlation, stepwise multiple regression and leave-one-out cross validation, was applied to investigate the relationship between landscape patterns and PM_2.5_ concentration. Specific details were provided as described in the following.

### PM_2.5_ measurements

**Fig 1:**
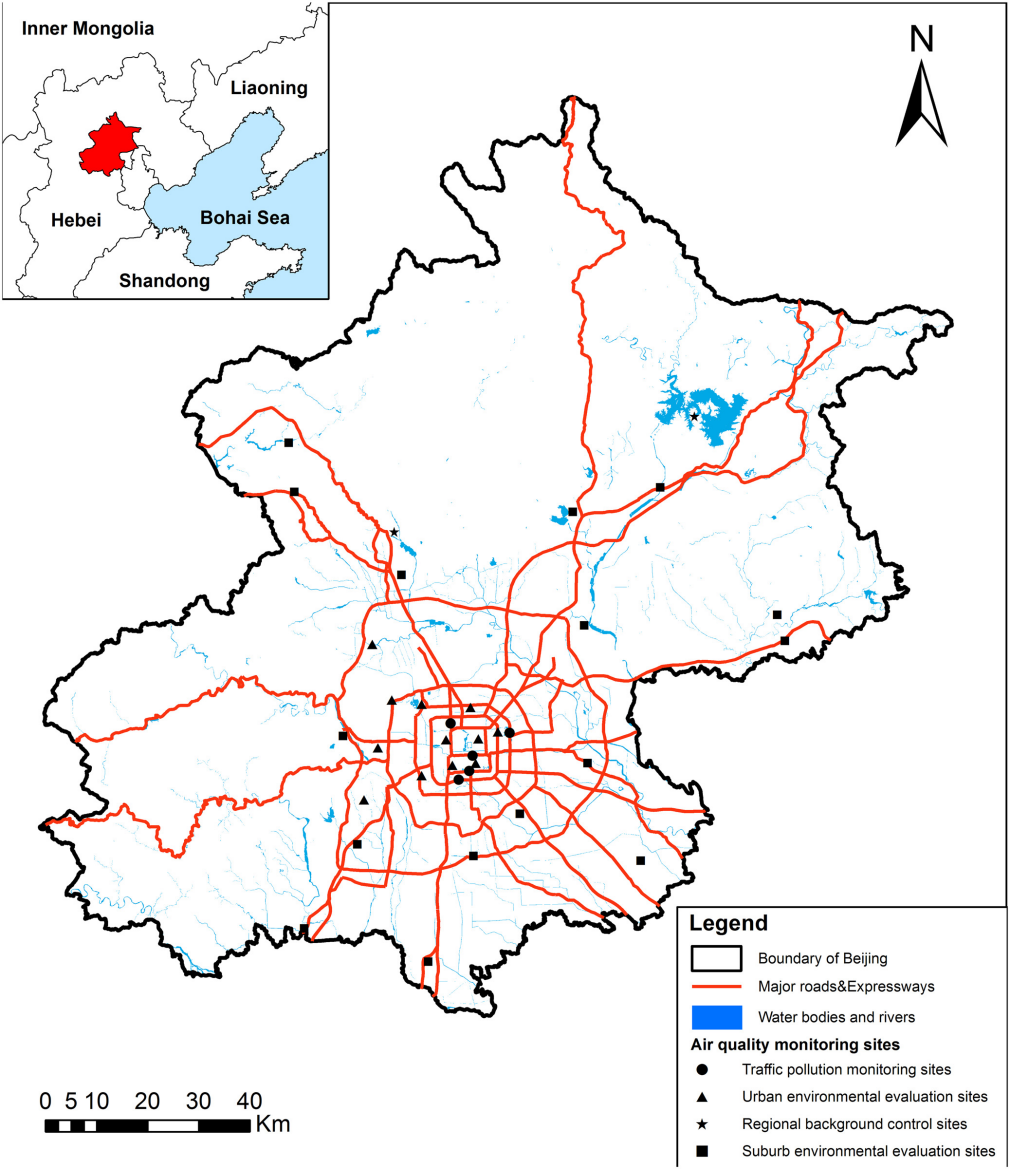
Classification and distribution of air quality monitoring sites in Beijing.

Routine monitoring data were collected at 35 air quality monitoring sites, which were available on the website of Beijing Environmental Monitoring Center in real time. Continuous hourly PM_2.5_ concentrations were measured for a whole year from 4th March 2013 to 8th March, 2014. The 35 sites were divided into four categories to guarantee adequate spatial variation in measured concentrations, including 12 urban environmental evaluation sites, 16 suburb environmental evaluation sites, 5 traffic pollution monitoring sites, and 2 regional background control sites. The distribution of all sites was illustrated as shown below (Fig 1).

### Land use data

Land use data for 2010 in Beijing were obtained from the Center for Earth System Science, Tsinghua University [47], derived from Landsat TM and ETM+ with a spatial resolution of 30m. While the original land cover data were classified into 20 land use categories, we simplified land use data into 5 categories: construction land, vegetation, water body, bare land and cropland for further analysis in landscape metrics variables.

### Landscape metrics

Based on the land use classification, landscape metrics of different level were calculated. Landscape metrics have been widely used to measure landscape patterns [48], which include composition and configuration [49]. According to former studies [32, 50, 51], 5 landscape metrics (Table 1) were selected to measure urban landscape patterns in Beijing based on principles including (1) theoretically and practically important, (2) easily calculated, (3) interpretable, and (4) little redundancy. The selected landscape metrics were applied to reflect the size, density, edge, shape of different patch types, and evenness and fragmentation of the whole landscape, respectively. The percentage of landscape (PLAND) is a landscape composition metric measuring the percentage of different patch types within the landscape. Patch density (PD), edge density (ED) are landscape configuration metrics describing the spatial distribution of patches within the landscape. Shannon’s evenness index (SHEI) and contagion (CONTAG) at landscape level signifies some characteristics of the whole landscape. Additionally, we adapted buffer radii of 100, 300, 500, 1000, 2000, 3000m and 5000m around the monitoring sites. The above metrics describing various patch and landscape types were calculated using Fragstats4.1 as variables.

**Table 1:** List of the selected landscape metrics.

Metrics (abbreviation)	Description (unit)	Range
Percentage of landscape (PLAND)	PLAND quantifies the proportional abundance of each patch type in the landscape (percent)	0 < PLAND ≤100
Patch density (PD)	PD expresses number of patches on a per unit area for considered class (number per 100 hectares)	PD > 0
Edge density (ED)	ED reports edge length on a per unit area for considered class (meter per hectare)	ED ≥ 0
Shannon’s evenness index (SHEI)	SHEI expresses the evenness distribution of area among patch types (none)	0 ≤ SHEI ≤ 1
Contagion(CONTAG)	Tendency of land use types to be aggregated (percent)	0 < CONTAG ≤ 100

Sources: Fragstats documents 4.2 (2014).

### Other variables

Other variables in this study also include street networks, population, and elevation etc. Street networks data were obtained through map vectorization from Google Earth combined with Landsat TM 2012. We categorized all roads into major roads and secondary roads and used the length of specific road types as traffic variables. Major roads include ring roads, expressways, and some other important roads, while secondary roads covered the rest. In accordance with the principle of traffic-related buffer selection described by Hoek et al. [52] We set the maximum traffic-related buffer distance to 1000m. Combined with dispersion patterns, we adapted circular buffers with 100, 200, 300, 500, 750, and 1000 m radii around the sampling sites.

Population dataset, from Institute of Geographic Sciences and Natural Resource Research, CAS, with a spatial resolution of 0.5′ (816.3m approximately), was used to represent the population distribution of Beijing. Considering the spatial resolution of the dataset, we only adapted buffer radii of 1000, 2000 and 5000m.

DEM data, derived from ASTER GDEM 1st edition (V1) with a spatial resolution of 30m, were obtained from Geospatial Data Cloud.

Land cover data, street networks, population density, and elevation were conducted in ArcGIS to develop the predictor variables for further study (Table 2).

**Table 2:** Classification and description of independent variables.

Class of variables	Description	Subclass of variables		Buffer radii(meters)	Variables names
Street network	The length of major roads and common roads within the buffer (m)	Mr (main roads)		100;200;300;500;750;1000	Mr_xx*
		Cr (common roads)		100;200;300;500;750;1000	Cr_xx
Population density	Population within the buffer(in units)	Pop (population)		1000;3000;5000	Pop_xx
Elevation	Elevation of the site (m)	DEM (elevation)			DEM
Landscape metrics	The landscape metrics of land use within the buffer	Crop (cropland)	PLAND	100;300;500;1000;2000;3000;5000	Crop_PLAND_xx
			PD	100;300;500;1000;2000;3000;5000	Crop_PD_xx
			ED	100;300;500;1000;2000;3000;5000	Crop_ED_xx
			SHEI	100;300;500;1000;2000;3000;5000	Crop_SHEI_xx
			CONTAG	100;300;500;1000;2000;3000;5000	Crop_CONTAG_xx
		Vege (vegetation)	PLAND	100;300;500;1000;2000;3000;5000	Vege_PLAND_xx
			PD	100;300;500;1000;2000;3000;5000	Vege_PD_xx
			ED	100;300;500;1000;2000;3000;5000	Vege_ED_xx
			SHEI	100;300;500;1000;2000;3000;5000	Vege_SHEI_xx
			CONTAG	100;300;500;1000;2000;3000;5000	Vege_CONTAG_xx
		Wat (water body)	PLAND	100;300;500;1000;2000;3000;5000	Wat_PLAND_xx
			PD	100;300;500;1000;2000;3000;5000	Wat_PD_xx
			ED	100;300;500;1000;2000;3000;5000	Wat_ED_xx
			SHEI	100;300;500;1000;2000;3000;5000	Wat_SHEI_xx
			CONTAG	100;300;500;1000;2000;3000;5000	Wat_CONTAG_xx
		Cons (construction land)	PLAND	100;300;500;1000;2000;3000;5000	Cons_PLAND_xx
			PD	100;300;500;1000;2000;3000;5000	Cons_PD_xx
			ED	100;300;500;1000;2000;3000;5000	Cons_ED_xx
			SHEI	100;300;500;1000;2000;3000;5000	Cons_SHEI_xx
			CONTAG	100;300;500;1000;2000;3000;5000	Cons_CONTAG_xx
		Bare (bare land)	PLAND	100;300;500;1000;2000;3000;5000	Bare_PLAND_xx
			PD	100;300;500;1000;2000;3000;5000	Bare_PD_xx
			ED	100;300;500;1000;2000;3000;5000	Bare_ED_xx
			SHEI	100;300;500;1000;2000;3000;5000	Bare_SHEI_xx
			CONTAG	100;300;500;1000;2000;3000;5000	Bare_CONTAG_xx

* xx corresponds to the circular buffer radii (in meters).

### Statistical analysis

Annual and seasonal average concentrations of PM_2.5_ in 35 sites were calculated as dependent variables in this study, while the independent variables are listed in Table 2. The statistical analysis includes Pearson’s correlation analysis, multiple stepwise regression, and leave-one-out cross validation (LOOCV). The method in this study was similar but not identical to LUR model. The LUR model, which was developed by Briggs et al. in 1997 [53]. It uses least squares regression to combine monitored data with GIS-based predictor data to build a prediction model applicable to nom-measured locations [54]. It usually contains these main steps, including obtaining the monitoring data, generating variables, developing models, model validation and regression mapping. The most prominent characteristic of LUR may be the predictor variables developed in GIS. Compared to LUR model, our study added landscape metrics as variables in the model, not only usual variables in other models, such as land use, street network et al., which did not occur in previous LUR models. In short, the difference between LUR models and our method was that we made use of landscape metrics to investigate the effect of landscape pattern on PM_2.5_ concentration more than land use factors.

In the first place, in order to conduct a tentative study on the effects of landscape patterns on PM_2.5_ concentration, Pearson correlation coefficients were applied to measure the relationship between them. Pearson correlation coefficient is a linear correlation coefficient used to reflect the extent of correlation between two variables. The larger the absolute value of correlation coefficient, the greater correlation they have [48]. This study made a Pearson correlation between each landscape metric and PM_2.5_ concentration, attempting to find the landscape metrics which have significant correlation with PM_2.5_ concentration (*P*<0.05). Pearson correlation analysis was performed to determine whether the landscape variables and PM_2.5_ concentration were highly correlated, which laid a foundation for further study.

Next, we tested the relationship between landscape patterns and PM_2.5_ concentrations by stepwise multiple regression. A multiple linear regression was performed using all variables in a stepwise selection method. The method selects a subset of the variables that have a high correlation with dependent variables. Only variables that made a significant contribution to the overall model were kept (*P*<0.05) [55, 56]. Stepwise multiple regression identifies which variables explain the greatest amount of variation in PM_2.5_ concentration. Before stepwise multiple regression, in order to avoid the potential of collinearity among variables belonging to the same category and ensuring interpretability of parameters, a model-building algorithm was used, as follows[19]: (1) Remove variables with less than five nonzero values; (2) in each sub-category, rank all variables by the absolute strength of their correlation with the measured pollutant and identify the highest-ranking variable; (3) remove other variables in each sub-category that are correlated (Pearson’s *r* > 0.6) with the highest-ranking variable; (4) enter all remaining variables into a stepwise multiple linear regression with a confidence interval 95% in SPSS; (5) remove the variables that have insignificant t-statistics (*P*<0.05) or are inconsistent with a priori assumptions; (6) repeat steps 4 and 5 until there are no more variables that can contribute less than 1% to the adjusted *R* ^*2*^. Then stepwise multiple regressions were performed to estimate the direction and magnitude of the effect of transportation, population density, elevation, and landscape pattern on PM_2.5_ pollution. There were 5 regression models in the study, including 1 annual average model and 4 seasonal average models.

In the last step, we evaluated the regression models by leave-one-out cross validation (LOOCV) [52], where models were developed for N-1 sites (N is the total number of sampling sites) and the predicted concentrations were compared with the measured concentrations at the left-out site. The above procedure was repeated 35 times. Then, the root mean squared error (RMSE) was calculated to describe the validity and accuracy of the models. Generally, a lower RMSE value meant more stable and accurate models.

## Results

### Descriptive statistics

**Fig 2:**
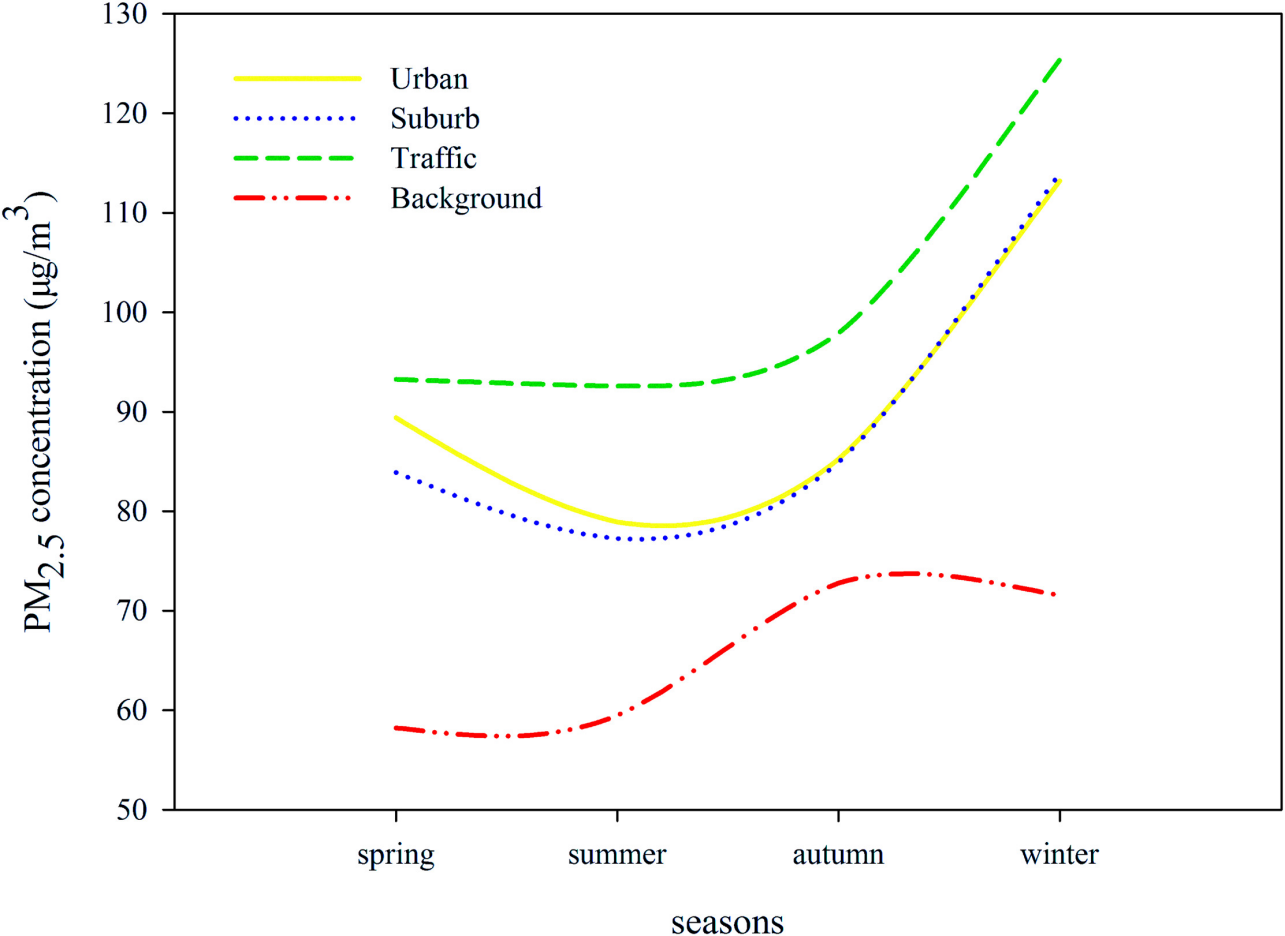
Seasonal pattern of four categories of all sites.

All 35 sites were valid samples during the study period. The annual average concentration of 35 sites was 90.724μg/m^3^, which was 2–3 times higher than the WHO Level 1 Interim Target of 35μg/m^3^. The maximum value of annual average concentration was 115.894μg/m^3^ in the Liulihe site, while the minimum value was 62.054μg/m^3^ in Miyun reservoir site. Fig 2 showed that PM_2.5_ concentration in winter and autumn was larger than that in spring and summer. The average concentration of four seasons was 85.349μg/m^3^, 79.149μg/m^3^, 86.887μg/m^3^ and 112.299μg/m^3^. The peak concentrations of spring, summer, autumn and winter were 109.930μg/m3 (Liulihe site), 100.899μg/m^3^ (Daxing site), 147.738μg/m^3^ (Daxing site) and 163.347μg/m^3^ (Inner YongDingMen street site). The PM_2.5_ concentration between different sites and different seasons changes greatly. The spatiotemporal variation of PM_2.5_ concentration in Beijing may be evident.

### Pearson correlation analysis

The relationship between PM_2.5_ concentration and landscape patterns was first characterized by Pearson correlation analysis. Pearson coefficients indicated the extent of the correlation. There were 4 landscape metrics which had a significant relationship (|*r*|>0.6) with PM_2.5_ concentration in Table 3. Firstly, among 5 land use types, only vegetation metric (vege_PLAND_5000) related closely with PM_2.5_ concentration. *R* values between vege_PLAND_5000 and annual, spring, summer, winter average concentration were -0.701, -0.790, -0.701 and -0.623 respectively, which indicated that increase of vegetation area could decrease PM_2.5_ concentration, especially in spring, summer and winter. Secondly, at class-level configuration metrics, only vege_ED_5000 had close correlation with PM_2.5_ concentration in spring (*r* = -0.776), summer (*r* = -0.776) and autumn (*r* = -0.612) particularly. Thirdly, at landscape level, both SHEI and CONTAG displayed a significant relationship to PM_2.5_ concentration, and they all belonged to the radii buffer of 3000. In addition, all metrics showed a negative relationship with PM_2.5_ concentration except CONTAG_3000. And all metrics at class-level had negative correlation with PM_2.5_ concentration, and they all related to vegetation, which was of great importance in mitigating PM_2.5_ concentration. Furthermore, from temporal perspective, the results of correlation in summer was more similar to that in annual average. They both had 4 metrics (vege_PLAND_5000, vege_ED_5000, SHEI_3000 and CONTAG_3000). While there were only 2 metrics in spring (vege_PLAND_5000 and vege_ED_5000), and was only 1 metric in autumn (vege_ED_5000) and winter (vege_PLAND_5000). From the correlation analysis, the metrics PLAND and ED of vegetation had significant relationship with PM_2.5_ concentration, which enhanced the importance of vegetation conservation. The SHEI and CONTAG also signified the effects of evenness on mitigating PM_2.5_ concentration. They laid a foundation for the further regression analysis.

**Table 3:** Landscape metrics that had relationship with PM_2.5_ concentration (|*r*|>0.6).

Class	Class-level composition metrics (r value)	Class-level configuration metrics (r value)	Landscape-level metrics (r value)
Landscape metrics (annual average)	Vege_PLAND_5000(-0.701)	Vege_ED_5000(-0.766)	SHEI_3000(-0.654)
			CONTAG_3000(0.631)
Landscape metrics (spring average)	Vege_PLAND_5000(-0.790)	Vege_ED_5000(-0.766)	
Landscape metrics (summer average)	Vege_PLAND_5000(-0.701)	Vege_ED_5000(-0.766)	SHEI_3000(-0.654)
			CONTAG_3000(0.631)
Landscape metrics (autumn average)		Vege_ED_5000(-0.612)	
Landscape metrics (winter average)	Vege_PLAND_5000(-0.623)		

### Stepwise multiple regression

To further analyze the contributions of landscape metrics to PM_2.5_ pollution in various seasons, stepwise multiple regression was employed and 5 regression models obtained. More details are shown in Table 4.

**Table 4:** Analysis of coefficient of regression models.

Regression model	Variables	Parameters of models			
		B	t	Sig.	
Year	Constant	90.962	14.956	0.000	Adjusted R^2^ = 0.849
	Vege_ED_5000	-0.428	-9.472	0.000	D-W value = 2.053
	Crop_PLAND_1000	0.347	5.402	0.000	RMSE = 4.754μg/m^3^
	Cons_PLAND_300	0.125	2.629	0.014	F = 32.819(Sig. = 0.000)
	Cons_PD_2000	-1.604	-3.094	0.004	
	Wat_ED_3000	-0.208	-2.966	0.006	
	Mr_1000	0.002	2.065	0.048	
Spring	Constant	90.767	63.759	0.000	Adjusted R^2^ = 0.802
	Vege_PLAND_5000	-0.485	-7.572	0.000	D-W value = 1.889
	Bare_ED_500	-0.464	-5.143	0.000	RMSE = 5.050μg/m^3^
	Wat_PLAND_5000	-0.530	-4.173	0.000	F = 46.933(Sig. = 0.000)
Summer	Constant	84.803	37.515	0.000	Adjusted R^2^ = 0.684
	Vege_PLAND_5000	-0.434	-5.504	0.000	D-W value = 1.849
	Vege_PD_5000	-0.698	-2.589	0.015	RMSE = 6.027μg/m^3^
	Mr_1000	0.005	3.788	0.001	F = 19.409(Sig. = 0.000)
	Wat_PLAND_500	-1.151	-3.207	0.003	
Autumn	Constant	90.819	24.020	0.000	Adjusted R^2^ = 0.624
	Vege_ED_5000	-0.289	-2.521	0.017	D-W value = 2.148
	Crop_PLAND_1000	0.528	5.010	0.000	RMSE = 10.317μg/m^3^
	Cons_PD_300	-0.853	-3.190	0.003	F = 12.288(Sig. = 0.000)
	Vege_PD_5000	-2.901	-3.214	0.003	
	Bare_PLAND_500	6.210	2.304	0.029	
Winter	Constant	45.596	2.288	0.029	Adjusted R^2^ = 0.658
	Vege_PLAND_5000	-0.873	-5.109	0.000	D-W value = 1.652
	Wat_ED_3000	-0.634	-4.513	0.000	RMSE = 12.956μg/m^3^
	CONTAG_3000	1.415	4.407	0.000	F = 22.805(Sig. = 0.000)

First, stepwise multiple linear regression of variables including street network, population, DEM and landscape metrics was performed to research the influence of urban landscape on PM_2.5_ concentration in the whole year. After removal of the non-significant variables, 6 significant variables (vege_ED_5000, crop_PLAND_1000, cons_PLAND_300, cons_ED_2000, wat_ED_3000 and mr_1000) were employed to correlate with PM_2.5_ concentration (Table 4). The obtained relationship is expressed by the following equation:

$$\begin{array}{l} PM_{2.5}{}^{year}\text{=}90.962-0.428\cdot vege\_ED\_5000+0.347\cdot crop\_PLAND\_1000+0.125\cdot cons\_PLAND\_300\\ -1.604\cdot cons\_PD\_2000-0.208\cdot wat\_ED\_3000+0.002\cdot mr\_1000 \end{array}$$ (1)

There existed negative linear correlation between PM_2.5_ concentration and vege_ED_5000, cons_PD_2000 and wat_ED_3000. A positive linear correlation of PM_2.5_ concentration between crop_PLAND_1000, cons_PLAND_300 and mr_1000 was found. The order of absolute coefficients suggesting that the correlations for cons_PD_2000 was closer than other 5 variables. The order of sequence into the regression model indicated that vege_ED_5000 was of most importance for decreasing PM_2.5_ concentration.

Next, according to different kinds of independent variables in different period, 4 models were obtained as follows:

$$PM_{2.5}{}^{spring}=90.\text{767}-0.\text{485}\cdot vege\_PLAND\_5000-0.\text{464}\cdot bare\_ED\_500-0.\text{53}0\cdot wat\_PLAND\_5000$$ (2)

$$\begin{array}{l} PM_{2.5}{}^{summer}=84.\text{8}0\text{3}-0.\text{434}\cdot vege\_PLAND\_5000-0.\text{698}\cdot vege\_PD\_5000+0.00\text{5}\cdot mr\_1000\\ -\text{1}.\text{151}\cdot wat\_PLAND\_500 \end{array}$$ (3)

$$\begin{array}{l} PM_{2.5}{}^{autumn}=90.\text{819}-0.\text{289}\cdot vege\_ED\_5000+0.\text{528}\cdot crop\_PLAND\_1000-0.\text{853}\cdot cons\_PD\_300\\ -\text{2}.\text{9}0\text{1}\cdot vege\_PD\_5000+\text{6}.\text{21}0\cdot bare\_PLAND\_500 \end{array}$$ (4)

$$PM_{2.5}{}^{winter}=45.\text{596}-0.\text{873}\cdot vege\_PLAND\_5000-0.\text{634}\cdot wat\_ED\_3000+\text{1}.\text{415}\cdot CONTAG\_3000$$ (5)

There were 3 variables appearing in the regression model of spring. From the perspective of coefficient, the order of the three variables was vege_PLAND_5000>bare_ED_500>wat_PLAND_5000, showing that the composition of vegetation and water had significant relationship with PM_2.5_ concentration, and the edge density of bare land also negatively related to PM_2.5_ concentration. In regression model of summer, 3 variables were landscape metrics among all 4 variables except mr_1000. The maximum absolute coefficient existed in wat_PD_500, which means it played the most related role in PM_2.5_ concentration. Additionally, there existed negative linear correlation between PM_2.5_ concentration and vege_PLAND_5000, vege_PD_5000 and wat_PLAND_500, with positive linear correlation of PM_2.5_ concentration and mr_1000. There were 5 variables in autumn model, bare land and cropland could increase PM_2.5_ in autumn, which contributed more in this season. In winter, the model showed what differently comparing to other seasons was CONTAG_3000, the only 1 landscape metrics at landscape level, indicating the feature of whole landscape also influence the particulate matter. Table 4 shows that the significance of regression coefficients t value and models *F* value were less than 0.05, indicating that each partial regression coefficient in the regression equation were significant, and each regression model was valid. The adjusted *R* ^*2*^ of these 5 regression equations was 0.849, 0.802, 0.684, 0.624 and 0.658 respectively. In the LOOCV, the RMSE for PM_2.5_ concentrations models were 4.754μg/m^3^, 5.050μg/m^3^, 6.027μg/m^3^, 10.317μg/m^3^ and 12.956μg/m^3^. The results was acceptable comparing to other studies [20, 22], especially the first 3 models.

On the basis of the above results, the classification of variables in each regression equation was made in Table 5, according to the different variable types. As shown in Table 5, transportation and landscape metrics were 2 kinds of main variables that entered into the stepwise multiple regressions. There were 9 class-level composition metrics in the overall models. 3 metrics belonged to vegetation, 2 metrics belonged to water body and cropland, and the rest was construction land and bare land. Class-level configuration metrics contained 9 metrics, including 4 vegetation metrics, 2 water body metrics, 2 construction land and 1 bare land metrics. Among these 5 models, only 1 landscape-level metric, CONTAG_3000 appearing in the model of winter. Besides, other dependent variables only contained transportation indicator, mr_1000. As for their effects on PM_2.5_ concentration, all configuration metrics at class-level had negative relationship with PM_2.5_, while CONTAG_3000 and mr_1000 related positively with PM_2.5_. However, the effects of composition metrics at class-level on PM_2.5_ varied with the landscape types. The composition of vegetation and water body had negative effect on PM_2.5_, but that of construction land, bare land and cropland had opposite influence. Furthermore, the variables also changed with the season, the most obvious one was crop_PLAND_1000 in model autumn, which indicated the crop was a special landscape types for air pollution. In conclusion, regression analysis made a further and more accurate results than correlation analysis. Different land use had different influence on PM_2.5_ concentration. Vegetation and water could deposit particulate matter, while bare land and construction land could produce particulate matter, crop land had uncertain relationship with PM_2.5_ concentration. As for landscape configuration metrics, both ED and PD may decrease PM_2.5_ concentration no matter what kind of land use. The evenness of whole landscape and main road contributed the PM_2.5_ concentration, too.

**Table 5:** Classification of independent variables included in regression models.

Classification	Class-level composition metrics	Class-level configuration metrics	Landscape-level metrics	Other variables
Model year	Crop_PLAND_1000(+)	Vege_ED_5000(-)		Mr_1000(+)
	Cons_PLAND_300(+)	Cons_PD_2000(-)		
		Wat_ED_3000(-)		
Model spring	Vege_PLAND_5000(-)	Bare_ED_500(-)		
	Wat_PLAND_5000(-)			
Model summer	Vege_PLAND_5000(-)	Vege_PD_5000(-)		Mr_1000(+)
	Wat_PLAND_500(-)			
Model autumn	Crop_PLAND_1000(+)	Vege_ED_5000(-)		
	Bare_PLAND_500(+)	Cons_PD_300(-)		
		Vege_PD_5000(-)		
Model winter	Vege_PLAND_5000(-)	Wat_ED_3000(-)	CONTAG_3000(+)	
Land use types (number of appearing in models)	Vegetation (3)	Vegetation (4)		
	Water body (2)	Water body (2)		
	Cropland (2)	Construction land (2)		
	Construction land(1)	Bare land (1)		
	Bare land (1)			

## Discussion

### Effects of urban landscape composition on PM_2.5_ concentration

The concentration of PM_2.5_ was controlled by multiple factors, such as wind, precipitation, traffic conditions etc., but this paper took major focus on urban landscape pattern. PLAND, the composition metric, characterizes the percentage of patch classification in the whole landscape [50], from which we could learn the effect of land use types on PM2.5 indirectly. More information can be gained in regression models. In 5 models, all land use types all entered. The land use types most frequently associated with PM_2.5_ concentration was vegetation, next were water body and cropland, bare land and construction land were least. The coefficients of vege_PLAND in model spring, model summer and model winter were -0.485, -0.434 and -0.873 respectively. The coefficients of wat_PLAND in model spring and model summer were -0.530 and -1.151 respectively. They were negative, indicating that increasing the sink landscape percentage would decrease PM_2.5_ concentration. Cons_PLAND (coefficient was 0.125 in model year), bare_PLAND (coefficient was 6.210 in model autumn) and crop_PLAND (coefficients were 0.347 and 0.528 in model year and model autumn) were proved to influence PM_2.5_ oppositely, for the coefficients were positive in models. In fact, it was easy to understand the role of PLAND on PM_2.5_. Sink landscape can absorb PM_2.5_, and construction land can produce particulate matter [57]. Altering their areas caused increasing and decreasing PM_2.5_, demonstrating results similar to many relative studies.

A number of previous studies have shown a strong relationship with land use, mainly applying LUR models [5, 22] and simple linear regression [18]. Those studies similarly showed that vegetation [27, 47] and water [22] were able to reduce PM_2.5_ concentration. As a sink landscape for PM_2.5_, vegetation and water play a primary role in PM_2.5_ pollution, as was derived from correlation analysis and stepwise multiple regression. Vegetation mainly absorbed particulate matter through leaves by dry and wet deposition to reduce ambient PM_2.5_ concentration. Tree planning has been put forward by the Beijing municipal government as a major measure to improve air quality. Yang et al. [58] used an urban forest effects model to explore the effect of urban forest on air pollution. Results showed that trees in central Beijing removed 1261.4 tons of pollutants, most of which were particulate matter. Research in 10 U.S. cities also showed similar results in that the amount of PM_2.5_ removed by trees ranged from 4.7 tons to 64.5 tons annually, for instance saving the state of New York 60 million dollars in healthcare costs and mortalities because of cleaner air [27]. Besides regression models, some studies based on physical model also showed the similar results. Gromke [59] indicated that the trees had bad influence on pollutant dispersion using a new vegetation model. Ji and Zhao [60] used drift flux model and Reynolds-Average Navier-Stokes (RANS) model to investigate the effect of trees on particle concentration distributions and building. Dzierzanowski et al. [61] further focused on the various functions of different tree species on particulate matter. These results of above studies all revealed that vegetation had the great impact on PM_2.5_ mitigation, which agreed with the results of this study. Further, water played a significant role as well, primarily depositing and absorbing particulate matter across vast surfaces, which complied with results in experiment and simulation studies [22, 62]. However, water’s effect may not obviously comparing with other land use types. So it has often been often neglected in LUR models [52]. Nonetheless, this study confirmed the significance of water, which should be given more attention in future study.

Besides water body and vegetation, cropland, construction land and bare land were also important land use type in regression models. Cropland was a special factor. On the one hand, as a part of vegetation [63], it can reduce PM_2.5_ concentration by deposition; on the other hand, smoke can be produced by straw burning during harvest, the reason that many cities reach peak PM_2.5_ concentration in autumn, which could be shown obviously in model autumn. In addition, cropland can be considered bare land when it is left aside. Thus, the effect of cropland on PM_2.5_ concentration depended on the balance between these two functions. So only in model autumn could crop_PLAND entered, which not only verified but highlighted the importance of cropland for air pollution, in autumn particularly. Meantime, construction land shared a mass of impervious surfaces. The rapid development of urbanization has led to increasing areas of construction land, bringing burgeoning population and growing transportation use [64], increasing the potential for PM_2.5_. According to Tan et al. [65], who took Taiwan as a case study for analyzing the holiday effect on air quality, low urbanization areas always had better air than cities in Taiwan, no matter what period of the year. As a consequence, during the process of urbanization, how to manipulate PM_2.5_ will be the most important issue to deal with.

Furthermore, there were some differences among four seasons. Firstly, among the four seasons, only autumn model contained the crop variable. Land use did not change greatly during the year, but crop was a special land use. In autumn, the crop could produce smoke by straw burning, either in Beijing or surrounding regions, which make it significant in autumn model rather than other three seasons. Secondly, the winter model only contained two variables, producing a lower *R* ^2^. It may be explained by specific PM_2.5_ sources that were distributed in a small scale, such as meteorological conditions, fossil fuel combustion, biomass burning for cooking and winter heating [17, 66–68], and setting off fireworks in the winter [69, 70]. Thirdly, bare land could be a source because of the soil or sand dust caused by wind erosion, especially in the spring of Beijing, when sand storm happened frequently. In conclusion, due to the regression algorithm, which only picked up the variables that were significant, and complicated influence of many other factors, it was not easy to explain all different variables between seasons.

### Effects of urban landscape configuration on PM_2.5_ concentration

The relationship between urban landscape patterns and PM_2.5_ concentration were not clearly noted in previous studies. The conclusion that landscape patterns correlated to PM_2.5_ concentration was obtained in our study through correlation analysis and stepwise multiple regression. Pearson correlation analysis (Table 3) showed that ED had a significant relation to PM_2.5_ concentration. Pearson coefficients between Vege_ED and PM_2.5_ concentration were -0.766 and -0.612 in different models. While in regression models, ED and PD were entered as well. Results revealed ED and PD had more evident relationship with PM_2.5_, which was rarely referenced in other studies.

In theory, according to the relationship between PLAND and PM_2.5_ concentration, the problem of PM_2.5_ could be dealt with by changing the percentage of specific patches, but there would be little possibility to increase sink landscape area or decrease construction land area in many quickly developing cities, especially in Beijing [46]. So the positive role of landscape configuration must be given full play in solving serious haze problems. Unfortunately, few studies have investigated the effect of configuration on PM_2.5_. Morani et al. [36] only discussed best tree planting locations to reduce the concentration of particulate matter. Zhang et al. [71] simulated the airflow and PM_2.5_ dispersion making use of large eddy simulation method, which took street canyon as a special land structure factor. The studies of Ji and Zhao [60] also provided suggestions on effective configuration for trees to reduce PM concentration. These regression and simulation results revealed that the configuration may related with pollutant to some extent. Correlation analysis and stepwise multiple regression results in our study showed that not only composition but also configuration affects air pollution. In other words, we can ease haze by transforming patch percentage and optimizing the configuration. It is noteworthy that numerous studies have focused on the relationship between landscape patterns and heat island. In fact, the haze island caused by PM_2.5_ was similar to heat island to some extent. Connors et al. [72] pointed out that the impact of configuration on urban heat island was context-dependent, and the most important metrics influencing it were LSI and ED. Buyantuyev and Wu [73] explored heat island and landscape heterogeneity. They learned that the interaction between land utilization and patterns of human activities will affect city temperatures. The relationship between configuration of various patches of urban landscape and PM_2.5_ concentrations can be explained by the following principles.

Both ED (edge density) and PD (patch density) can be used to represent the complexity of the edge and reflect the degree of interaction between certain landscape and ambient landscapes, Vege_ED, wat_ED, bare_ED, cons_PD, cons_PD and vege_PD in models all appeared to be important for explaining variation in PM_2.5_ concentrations. Increasing ED and PD of these land use types can mitigate PM_2.5_ more efficiently based on our study. It could be explained by the following reasons. Firstly, along with increasing ED and PD, the interaction between land use types can be more intensive, playing a more useful role for vegetation in PM_2.5_ deposition. Vegetation can absorb more particulate matter produced from other source landscapes. Secondly, urban landscape configuration could affect ambient microclimate, such as wind, humility and temperature et al. Canyon effect was a focus researching the relationship between urban structure with climate and human activity [32]. The street structure could change wind speed and direction. Heat island was largely influenced by urban configuration, indicating the effect of urban landscape pattern on temperature [74]. These methodology conditions were the main factors influencing PM_2.5_. In result, ED and PD could affect the ambient air pollution in an indirect way. Lastly, the edge and patch density may change the human activity, which may be another reason for mitigating PM_2.5_ concentration. However, the increase of edge density and patch density may enhance energy flow and exchange between green land and surrounding patches, therefore depositing more PM_2.5_ from its ambient area, which leads to reducing the PM_2.5_ concentration [75]. Edge and patch density availability can optimize the configuration of sink landscapes, creating more opportunities for source landscape and sink landscape to interact, enhancing particulate pollution removal. This is of great significance for urban landscape planning and management.

### Effects of whole landscape patterns on PM_2.5_ concentration

The results from our study also revealed that landscape-level metrics can affect PM_2.5_ concentration as well as class-level metrics. We chose SHEI and CONTAG to reflect the evenness and fragmentation of the whole landscape. SHEI had significant negative correlation with PM_2.5_ concentration. In correlation analysis, r value between SHEI_3000 and PM_2.5_ concentration was -0.654, while the CONTAG_3000 (r = 0.631) showed a positive relation to PM_2.5_ concentration. In regression models, CONTAG_3000 was shown to effect PM_2.5_ concentration in winter. Therefore, at the landscape level, SHEI and CONTAG were two factors influencing PM_2.5_.

SHEI can reflect the landscape heterogeneity of patches types, and it is sensitive to the distribution of various patches. The larger index suggests the more well-distributed landscape. The more well-distributed the landscape, the closer the relationship between each land use and more interaction between “sink” landscape and “source” landscape have, thus more frequently mitigating the PM_2.5_ pollution [46]. CONTAG describes the tendency of land use types to be aggregated. The smaller index means more scattered landscape, which indicates there are many different small patches. In other words, the communication between them can be more effective [35]. On the other hand, humility and heat also varies with the whole landscape pattern. This theory was the same as that relating to ED and PD. As a consequence, we should try to evenly distribute all kinds of patches in the whole landscape and balance the source landscape functions and sink landscape as much as possible. The findings from our study that both class-level and landscape-level metrics influenced PM_2.5_ concentration is of significance to urban landscape planning and management.

### Limitations and recommendations for future studies

There were data from only 35 sites analyzed in this study due to number of monitoring sites in Beijing, which could reduce the precision of regression equations to a certain extent. The ideal number of sites is 40–80, according to Hoek et al. [52]. Moreover, air pollution data was influenced by time and location, so it was difficult to assess the timeliness and stability of the results. It was no doubt that PM_2.5_ was controlled by many factors besides the variables in our study. Xie et al. [76] found that PM_2.5_ concentration had relationship with SO_2_, NO_2_, CO and O_3_ according to the case studies in 31 Chinese cities. Wu et al. [17] added canteen amount as an independent variable in Beijing study. The recent research took meteorological factors into account, such as humidity, wind speed and wind direction [77,78]. Tang et al. [79] considered the influence of street pattern and building height. With the rapid development of technique and data sharing around the world, there would be more potential variables adding to the models. The selection of variables in our study partially due to the inaccessibility of more data. Furthermore, both adjusted *R* ^2^ and RMSE, which usually used to describe the performance of model, were among reasonable range comparing to others regression models [52]. So the results in this study could be also useful for estimating the effect of landscape features on PM_2.5_ concentration though without considering other factors.

The results from our study verified that urban landscape pattern could also affect PM_2.5_ concentration, which may be of some benefit for air pollution management and landscape planning. However, the mechanisms and processes responsible for the effects of landscape metrics on PM_2.5_ pollution and seasonal differences could not be identified clearly from the statistical models applied in this study. In addition, it may be difficult to expect the performance of the models adding more variables, but such experiment would definitely be in our further study. Other more research, such as spatiotemporal characteristics of the effects, the impact scale and intensity, and mechanisms of seasonal differences were also included.

## Conclusions

There is no doubt that PM_2.5_ has become a serious air pollution problem in many rapidly developing cities. PM_2.5_ not only harms the environment, but also harms human health. So how to mitigate PM_2.5_ concentration is a focus of much research. Taking Beijing, one of the most air-polluted cities in China, as an example, our study quantitatively investigated the effects of urban landscape patterns on PM_2.5_ concentration using correlation analysis and stepwise multiple regression. Our study yielded the following conclusions:

1. Among landscape composition, as sink landscape for PM_2.5_, vegetation and water body had the most obvious influence on PM_2.5_ concentration. Vegetation and water can absorb particulate matter to reduce pollutants, while construction land and bare land will increase the concentration, and cropland was a special factor for its different function varying with seasons.
2. Configuration metrics at class-level ED and PD were significantly related with PM_2.5_ concentration. The larger ED and PD of landscape would remove more PM_2.5_.
3. Landscape-level metrics influenced PM_2.5_ concentration as well as class-level metrics. The evenness (SHEI) and fragment (CONTAG) of the whole landscape had a significant relationship with PM_2.5_ pollution. More even and scattered landscape distribution may be better for mitigating the particulate matter.

The above results can provide additional useful information for better urban landscape planning and management.
